# Continuous improvement towards environmental protection for pharmaceuticals: advancing a strategy for Europe

**DOI:** 10.1186/s12302-025-01180-z

**Published:** 2025-07-26

**Authors:** Sam Harrison, Catherine Barnett, Stephen Short, Cansu Uluseker, Patricia V. Silva, Maria D. Pavlaki, Sarah Roberts, Madalena Vieira, Stephen Lofts, Susana Loureiro, David J. Spurgeon

**Affiliations:** 1 UK Centre for Ecology & Hydrology, Library Avenue, Bailrigg, Lancaster, LA1 4AP UK; 2 UK Centre for Ecology & Hydrology, Benson Lane, Crowmarsh Gifford, Wallingford, OX10 8BB UK; 3 CESAM- Centre of Environmental and Marine Studies & Department of Biology, University of Aveiro, Aveiro, Portugal

**Keywords:** Pharmaceuticals, Environmental risk assessment, Pharmaceutical legislation, Ecotoxicity, Environmental exposure

## Abstract

The manufacture, use and disposal of pharmaceutical products can lead to their release into the environment, raising concerns about potential risks to biota and human health. This is recognised in the European Commission’s Pharmaceutical Strategy for Europe, which has recently overseen the development of a proposed revision of current pharmaceutical legislation. Amongst other things, this strategy and revision broadly offer strengthened protection against environmental risk. For example, it enhances the power authorities have to refuse market authorisation where an identified risk to the environment cannot be sufficiently addressed, includes a requirement for legacy pharmaceutical products to be risk assessed, includes a broadened scope to cover the entire product lifecycle, and places a greater focus on open data. In this publication, we assess the proposed legislation against the latest scientific knowledge, identifying opportunities for strengthening the protection that it offers. These opportunities include moving towards the risk assessment of mixtures, broadening the scope of effects testing to include non-conventional (e.g., behavioural) and chronic endpoints, making better use of predictive modelling such as Quantitative Structure–Activity Relationships (QSARs), and better accounting for environmental heterogeneity, such as the influence of temperature and especially pH on risk. Many of the elements discussed apply not just to pharmaceuticals but across other chemical domains and regulatory regimes, particularly when considering mixture assessment. Integrating knowledge across sectors and regulatory regimes is therefore crucial to better address the role of pharmaceuticals as one of many environmental pollutants.

## Introduction

The manufacture, use and disposal of human and veterinary pharmaceutical products can lead to their release into the natural environment, with use being the greater contributor to these releases, followed by disposal and then manufacturing [1]. This raises concerns about potential risks to biota and human health [2, 3]. Pharmaceutical regulation is, amongst other things, responsible for ensuring that the environmental impacts and risks resulting from medicine use are thoroughly assessed to inform appropriate risk mitigation measures. This is often achieved through the requirement for manufacturers to complete an environmental risk assessment (ERA) when they submit new pharmaceutical products for market authorisation [4]. An ERA entails assessing the behaviour of pharmaceutical compounds when they enter the environment, including their fate, exposure and effects. For this regulation to be effective, it is important that the guidance that underpins it—for example, guidance dictating how the ERA should be performed—keeps pace with evolving scientific understanding of current pharmaceuticals in the environment and emerging risks posed by new pharmaceutical products. However, despite significant scientific progress in recent decades to better understand the fate, effects and risks of pharmaceuticals [5–7], updates to regulatory guidance are often slow and limited in scope. In this paper, we discuss such regulation in the European Union (EU) context, identifying recent changes as a high-level perspective by which to highlight further opportunities for scientific advances to be embedded within it.

**Fig. 1:**
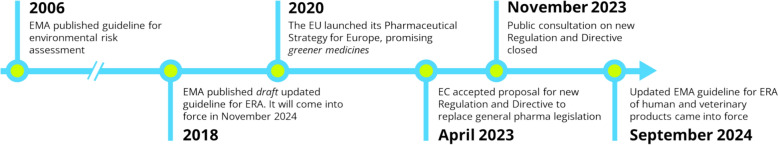
Timeline showing key points in the history of pharmaceutical legislation in the EU since 2006

Figure 1 shows a timeline of significant events regarding pharmaceutical legislation in the EU since 2006. Until recent years, regulation on the risk assessment of pharmaceuticals in the EU has remained essentially unchanged over the past two decades. Notably, until September 2024, the guideline covering the ERA of medicinal products for human use was that initially published by the European Medicine Agency (EMA) in 2006, with the only updates since its original enforcement being confined to clarification question and answer documents [8].

In 2018, a new draft guideline for the ERA of medicinal products was released, which, after a consultation and revision process, was finalised in February 2024 and came into force in September 2024 [4]. During this period, the European Commission also launched its Pharmaceutical Strategy for Europe [9], which, in 2023, resulted in the publication of a proposed new Directive [10] and Regulation [11] to replace the existing general pharmaceutical legislation on pharmaceuticals for human use (see Box 1). The scope of these instruments is broader than ERA, but a key objective is to make pharmaceuticals more environmentally sustainable. As such, many elements of the legislation target reducing environmental risk. Public consultation on the Directive and Regulation ended in November 2023, and the responses to this consultation are currently being reviewed by the European Commission.

Both the new guidance and strategy can broadly be seen as offering strengthened protection for the environment (and, therefore, indirectly humans via the environment). However, it would be remiss not to assess whether they provide protection commensurate with the latest scientific knowledge; in particular, whether there are gaps that could mean potential sources of environmental risk are missed. Several publications in the last 15 years have discussed gaps in current regulation and scientific knowledge [5–7, 12–14], and Whomsley et al. [15] have already provided an assessment of the new EMA guideline. Therefore, this publication focuses on the proposed Directive and Regulation. After describing our methodology, we begin by offering a summary of changes relevant to environmental risk and discuss the impact of these changes on the pharmaceutical sector, including manufacturers and users. We then discuss several areas where we believe strengthened environmental protection could be beneficial and is sufficiently supported before concluding with a discussion on the feasibility of integrating strengthened protection into regulation. The Directive and Regulation specifically cover medicinal products for human use, and therefore human use is the focus of this article, though many of the elements discussed are equally as relevant for veterinary medicines.

Box 1: EU Directives and Regulations Legislation in the EU is comprised mainly of Regulations and Directives. These are both legal acts but function in subtly different ways:

A* Regulation* is a binding legislative act that applies to all member states, without needing to be transposed into national law. It is enforceable as law simultaneously across all member states, with no room for variation. One of its main advantages is that it ensures uniformity across the EU.

A* Directive* is a legislative act that sets out a goal for member states to achieve but does not define how member states transpose this goal into national law. It is therefore up to member states to define their own national law, usually within a timeframe set out by the European Commission. A key advantage of Directives is that they achieve a unified result across member states but give flexibility to member states to implement these as best they see fit within their own legal system.

## Methodology

The evidence presented in this publication was compiled using a multifaceted approach, considering relevant legislative documents, associated literature (including grey literature) and assessing relevant initiatives and projects.

More specifically, the review of the differences between the EMA guideline [16] and the proposed regulations [10, 11] related to ERAs was conducted in September 2023. Various other sources of information were also consulted [17–28]. All the information related to ERAs presented in these documents was then collated and summarised; the most notable differences are presented in Table 1.

**Table 1:** The main differences between the current and proposed new pharmaceutical legislation that are related to environmental risk assessment (ERA)

Current legislation	Proposed legislation
Only use, storage and disposal phases are in scope [4, 34]	Environmental impact must be assessed across the entire product lifecycle, including manufacture (which may occur outside of the EU)
Outcome of ERA cannot be used as basis for refusal of market authorisation	Authorities can refuse, suspend or vary an authorisation based on environmental risk that cannot be mitigated
Products that entered the market pre-2006 do not need ERA	ERA must be carried out on products registered pre-2006, within 30 months of the new legislation coming into force
Specific labelling requirements for substances that are persistent, bioaccumulative and toxic (PBT) or very persistent, very bioaccumulative (vPvB). No requirements for mobility (PMT/vPvM) or endocrine disrupting labelling	Substances that are identified as PBT/vPvB, PMT/vPvM or endocrine active agents must be identified
Antimicrobial resistance is out of scope	Significant focus on the potential for antimicrobial resistance, with the requirement for a “stewardship plan” which includes information on risk mitigation measures, monitoring and reporting of resistance to the medicinal product

*PBT/PMT* Persistent, bioaccumulative/mobile and toxic, *vPvB/vPvM* Very persistent and very bioaccumulative/mobile

The analysis of the public consultation process [29] involved collecting stakeholder feedback through multiple consultation periods between 2021 and 2023. All feedback responses were systematically reviewed and filtered based on explicit references to "Environmental Risk Assessment (ERA)" and "environment". The relevant comments were identified, categorised, and analysed to pinpoint common concerns, key themes, and suggested improvements. Responses without direct relevance to ERA or environmental concerns were excluded.

## Proposed regulation and directive

In 2020, the EU launched its Pharmaceutical Strategy for Europe [9]. The broad aim of this initiative was to create a “future-proof regulatory framework” following an approach that covers pharmaceuticals’ entire lifecycle that, through one of its four central pillars, supports EU industry competitiveness, innovation and sustainability. It builds on the 2019 strategic approach of Pharmaceuticals in the Environment (PiE) [30] and aligns with several other EU initiatives, such as the European Industrial Strategy [31], the European One Health Action Plan [32], and notably for this publication, the European Green Deal [33].

As part of this strategy, in 2023, the European Commission adopted a proposal to revise and replace the existing general pharmaceutical legislation (Regulation 726/2004, Directive 2001/83/EC, Regulation 1901/2006, Regulation 141/2000/EC), which comprises various Regulations and Directives dated from 2000 to 2006. The revised legislation is formed of a Regulation and Directive, whose main objectives relate to those of the Pharmaceutical Strategy for Europe. The first two objectives are to ensure patients have timely and equitable access to safe, effective and affordable medicines via robust and secure supply chains. The third is to ensure the EU is an attractive and innovation-friendly environment for industry. Most relevant here are the final two objectives, which specifically relate to the environment:

- Make medicines more environmentally sustainable;
- Address antimicrobial resistance (AMR) and the presence of pharmaceuticals in the environment through a One Health approach.

The new proposal was open for public consultation for two periods in 2021 (March to April and September to December) and again from April 2023 to November 2023. During the latter period, 122 unique responses were submitted to the Regulation and 254 unique responses to the Directive, alongside numerous commentary pieces from various organisations, both giving positive and negative appraisals [17, 24–28]. Sect. "Strengthened environmental protection" discusses the main changes proposed under this legislation from an environmental risk perspective and Sect. "Consultation responses" presents a summary of the stakeholder consultation.

### Strengthened environmental protection

Broadly speaking, the new legislation can be seen as strengthening environmental protection across various areas, but with potential impacts on manufacturers. The most notable changes between the current and new legislation are summarised in Table 1, and are discussed in more detail in this section.

#### Refusal of authorisation based on environmental risk

Pre-authorisation requirements are suggested in the proposed legislation. It reinforces the current mandatory ERA requirement for introducing products into the EU market. However, for the first time, if an ERA is “not adequate”, risk mitigation measures are either not implemented or are inadequate to address the identified environmental risk, or the public benefit–environmental risk profile is not favourable, then the EU authorities could refuse, suspend, or vary an authorisation based on the risk of environmental harm. A time limit will be set for the submission of missing data and if the information is not received within that time limit, the application will be considered withdrawn. This is a significant change from the current legislation, under which the potential for environmental risk does not provide grounds for a refusal of market authorisation. The legislation offers no guidance on assessing the benefit–risk profile, though there is a existing EMA guideline on performing such an evaluation for veterinary medicinal products [35], which could be used as a framework for human pharmaceuticals.

#### Post-authorisation risk assessments

Products authorised before 30th October 2005 will likely not have had an ERA performed [12]. Indeed, a recent study concluded that environmental data is lacking for more than half the pharmaceutical products present on the German market with predicted concentrations where environmental risks might arise (i.e. predicted environmental concentrations, $PE{C}_{\text{market}},$ greater than the Phase I surface water threshold of 0.01 µg/L) [36]. The proposed legislation includes a requirement for ERAs to be conducted on these products within 30 months of the legislation coming into effect. Considering the number of products that this covers—over 700 between EU central and national authorisation databases [37]—this presents a significant burden on the industry and raises questions about the responsibility for performing these ERAs within the given timeframe, as discussed further in Sect. 2.3. It is worth noting that, unlike other EU regulatory frameworks such as REACH (Registration, Evaluation, Authorisation and restriction of CHemicals), there are no production volume thresholds proposed in the new legislation. However, the ERA guideline [4] includes a predicted environmental concentration (PEC) action limit, below which it can be concluded that environmental risks are negligible, which results in a similar effect to that which the specification of production volume thresholds achieves; pharmaceutical products with very low production volumes require no further assessment or mitigation. However, this action limit does not apply to products with specific mechanisms of action—namely antibiotic, antiparasitic or endocrine active substances—which require tailored testing or a specific assessment strategy, regardless of the PEC.

Previous guidance stipulates that type II variations (variations which may have a significant impact on the quality, safety or efficacy of the medicinal product) or general extension applications that might lead to an increase in exposure, for example through “significant increase in the extent of use”, require an updated ERA [16]. This implies that new information solely relating to hazard did not require a re-assessment. The September 2024 revision of this guidance [4] strengthened this to state that an updated ERA is required “if new data emerge in the post-authorisation phase that require an update to the ERA” and further includes a requirement to perform and include with the ERA a targeted data search to identify endpoints of significance to the ERA from scientific literature. The proposed legislation reinforces this, indicating that market authorisation holders should “take account of scientific and technical progress” and update ERAs “without undue delay”. Though it is not yet defined what “without undue delay” means in practice, or how significant of a change to ERA conclusions requires an update, this could be interpreted as a move towards stricter post-authorisation update requirements. Pharmaceuticals in the environment has been a very active research field for many years, with joint public-industry funding through the Innovative Health Initiative leading to projects such as iPiE [38] and PREMIER [39], and EU Horizon Europe funding leading to the joint forces of several large international projects forming the so-called Green Pharma cluster [40]. Such projects are generating new information on hazards and exposure that companies will need to consider in their risk assessments.

#### Broadening of scope

For the first time, the proposed new legislation requires an assessment of the environmental impact of the entire product lifecycle, including manufacturing. Previous legislation required the assessment of environmental risk only during use, storage and disposal. A key impact of this change is that registrants must assess the potential environmental risk from manufacturing waste streams that lead to discharges into the environment, such as via wastewater effluent discharges to watercourses and biosolid amendments to land. This brings it in line with other global regulation such as the US Environmental Protection Agency’s Pharmaceutical Manufacturing Effluent Guidelines [41], and is commensurate with other initiatives, such as the AMR Industry Alliance’s Antibiotic Manufacturing Standard, which aims to minimise the risk of AMR in the environment resulting from the manufacturing of human antibiotics [42]. Applicants must demonstrate that these releases do not lead to adverse environmental risks, for example, from the release of active pharmaceutical ingredients (APIs) or byproducts of API synthesis. This may lead to longer timelines for product development, and investment may be required to identify less harmful ingredients or remediate waste prior to discharge. An added complication is that this manufacture may occur outside the EU but must still be considered. Failure to comply with the requirements could result in penalties and reputational damage.

Though the legislation does not explicitly mandate a full lifecycle (sustainability) assessment, this is a clear move towards, in the words of the Regulation (European Commission, 2023c), “more of a life cycle approach to authorising medicinal products”. A natural consequence of this is that it becomes more pertinent that manufacturers include environmental risk as an impact category in their lifecycle assessments (LCAs), noting that ecotoxicity and environmental risks have historically been absent or poorly integrated into pharmaceutical LCA [43–45]. In doing so, this enables a holistic assessment of how “sustainable” a product is across different potential environmental impacts (e.g., climate change, raw material usage, environmental risk). Delving into the intricacies of LCA is beyond the scope of this paper. Still, it is worth noting that there are several initiatives and projects ongoing in this area, largely aiming to standardise LCA, including the addition of an environmental risk aspect [40, 46–49].

Specific labelling of products is another key change included. Any products (or their ingredients/constituents) containing PBT (Persistent, Bio-accumulative and Toxic), vPvB (very Persistent and very Bio-accumulative), PMT (Persistent, Mobile and Toxic), vPvM (very persistent and very mobile) or endocrine-disrupting chemicals should be identified in ERAs and risk mitigation measures include removing or limiting emissions to air, water and soil (and human health for PBT substances) for the relevant products. Under the current ERA Guidance [4], only PBT/vPvB substances are required to be labelled.

The new mandate includes a significant scope extension to assess antimicrobial resistance (AMR). Such risk is to be evaluated for both human health and the environment, covering the use and disposal across the entire manufacturing supply chain, both inside and outside the EU. Manufacturers of new antibiotics are required to submit a “stewardship plan” that includes monitoring the development of AMR post-authorisation. The proposed legislation includes the introduction of measures to encourage the innovation of novel antimicrobials to avoid the development of resistant bacteria (and fungi). It is well established that certain pharmaceuticals, most notably antibiotics and antifungals, but also some active ingredients with other uses, can cause an increase in antimicrobial-resistant genes in microbial populations when released into the environment [50]. The presence of these resistance genes in environmental microbial populations may act as a reservoir of constructs that can be transferred to pathogenic microorganisms, emerging as antibiotic resistance in medical settings, making environmental AMR relevant to this issue. Assessing the risk of increasing AMR has been suggested to be included in the derivation of Environmental Quality Standards [51], and the AMR Industry Alliance has created a British Standards Institute–backed manufacturing standard to minimise the risk of AMR arising from manufacturing [42]. However, at present, assessing the risk of increasing AMR is not yet a routine part of any authorisation assessment, presenting a challenge in implementing this new guidance. The topic of AMR in the environment is broad and has already received much attention in the literature [50, 52–56], as well as governmental action to tackle the issue (e.g. the UK government's 20-year vision for AMR [57, 58]). This extensive body of work sets out many of the scientific and management challenges relevant to the AMR issue.

#### A move towards open data

Reflecting the potentially onerous nature of performing ERAs, and the potential for duplicated efforts for performing ERAs on different products with the same APIs (which require separate market authorisations), the new legislation makes a significant step towards open data.

First is the proposal to create an active substance-based review system of ERA data for authorised medicinal products—so-called “ERA monographs”. It is proposed that an ERA monograph should include a comprehensive set of physicochemical, fate and effect data, and that the system should be based on a risk-based prioritisation of active substances. Under the Directive, information, studies and data may be requested from the member states’ competent authorities and marketing authorisation holders during the preparation of the monographs. In cooperation with the member states’ competent authorities, a proof-of-concept pilot study is proposed that would be completed within three years of the legislation entering into force. Whilst this is a welcome step towards transparent and reusable data on pharmaceuticals, the scope for integrating knowledge and data from scientific publications is unclear. The ERA monograph system implies that monographs will only be developed during the market authorisation process and, therefore, potentially only include studies relevant to the market authorisation itself. There is no clear route for the inclusion of other scientific knowledge, such as research studies published after market authorisation or studies on pharmaceuticals not (yet) submitted for market authorisation into these holdings.

A second proposed aspect is to create and maintain a register of ERA studies, unless such information is made public by different means (e.g., potentially within the European Medicines Agency’s European Public Assessment Report). Information within this register would be publicly available unless commercially confidential. For setting up the register, market authorisation holders and competent authorities may be requested to submit results from studies already completed for products authorised within 24 months of the Directive entering into force.

The combination of ERA monographs and register of ERA studies significantly impact data sharing, both to expedite performing ERAs and to ensure that ERAs are based on as broad and representative a selection of data as possible. This move towards open data could positively impact other areas of pharmaceutical manufacture, such as LCA: Lack of data has long been noted as a barrier to accurate LCA of pharmaceutical products [44]. At the same time, environmental risk is often not included as an impact category in LCA [43, 45]. Monographs of API physicochemical, fate and effects data, alongside registers of openly available ERA studies, could significantly ease the integration of environmental risk into LCA.

Initiatives under the Pharmaceutical Strategy for Europe, such as the monograph and register of studies, should not be isolated from the European Commission's “one substance, one assessment” proposal [59]. This aims to reduce the burden of performing chemical assessments across different regulatory regimes by sharing knowledge and data. Chemicals used as pharmaceuticals may also be subject to separate regulations as, for example, pesticides or biocides, and could, therefore, benefit from such an approach. Recent work [60] has undertaken a mapping of chemicals across EU’s legal frameworks towards this approach, highlighting issues such as a lack of common chemical identifiers, replication of effort across, and an underestimation of this overlap across frameworks. The one substance, one assessment proposal includes the creation of a “Common Data Platform” as a “one-stop shop” for access to data on chemicals. Still, the proposed pharmaceutical Regulation and Directive make no mention of this. Linking the Pharmaceutical Strategy for Europe and the “one substance, one assessment” proposal will be crucial to truly streamline assessments across regulatory regimes and reduce the burden on manufacturers.

### Consultation responses

The consultation process yielded diverse feedback, with a strong focus on ERAs [29]. In the initial consultation (March–April 2021), stakeholders reviewed the “Likely Environmental Impacts” section of the Inception Impact Assessment, providing 173 responses. A broader public consultation (September–December 2021) gathered 478 responses, with significant contributions from Germany (18.2%), Belgium (16.7%), and France (9.2%). Notably, 48.1% of respondents were on the EU Transparency Register, indicating substantial engagement from recognised organisations. Throughout 2023, the Commission and Proposal Adoption phases received 254 and 112 responses, respectively. Feedback consistently underscored the importance of comprehensive ERAs across the pharmaceutical lifecycle, advocating for stricter legal requirements and enhanced environmental monitoring.

There was strong advocacy for ERAs covering the entire lifecycle of pharmaceuticals, including manufacturing, usage, disposal, and wastewater management, as highlighted by entities such as the German Environment Agency (Feedback Reference number: F2245388, 2021) and Novo Nordisk (F2252373, 2021). BUND/FoE Germany (F2242242, 2021) emphasised the importance of establishing clear and stringent legal mandates for ERAs, advocating for the reassessment and market withdrawal of environmentally harmful products, particularly those approved before 2006.

Additionally, there were widespread recommendations for enhanced monitoring of pharmaceutical substances and antibiotic resistance, accompanied by incentives for developing environmentally friendly pharmaceuticals—a concept referred to as "Green Pharmacy". Stakeholders also proposed stronger environmental control standards in pharmaceutical production and recommended targeted training for healthcare and agricultural professionals to promote sustainable pharmaceutical practices. Additionally, AMR emerged as a related critical concern, emphasising the need for more judicious use of antimicrobials and improved waste management practices to mitigate environmental pathways contributing to AMR.

Concerns were notably raised about balancing environmental protection against patient access and health, with stakeholders such as GSK (F3442794, 2023) and Bayer (F3442784, 2023) advocating for a balanced approach to avoid limiting access to essential medicines. It is worth mentioning that the legislation gives no guidance on where this balance of environmental protection against public benefit lies, and how any new requirements would ever lead to denial of market authorisation. Furthermore, Teva Pharmaceuticals (F3443059, 2023), EUCOPE (F3435862, 2023) and Angelini Pharma (F3442143, 2023) highlighted that heightened compliance requirements could disproportionately affect smaller companies, potentially leading to delays in product approvals and increased costs for such firms.

Another significant issue raised by responders such as APOGEN (F3437282, 2023), and Viatris (F3442928, 2023) was the potential for delays and duplication of efforts, particularly concerning generic and legacy medicines. AESGP (F3430600, 2023) highlighted the need for a streamlined process, advocating for data-sharing mechanisms to avoid unnecessary duplication of environmental assessments.

Overall, there was a general consensus among respondents on the need for a wider regulatory alignment by integrating the pharmaceutical ERA regulations with broader EU environmental policies, such as the European Green Deal and the Chemicals Strategy for Sustainability.

## A call for continuous improvement

The prospect of strengthened environmental protection offered by the new legislation was broadly recognised as a welcome step forward, despite challenges and nuances. It already begins to address gaps highlighted over the past decade [5, 6, 13], such as covering a broader range of pathways to exposure by considering full product lifecycles. In this section, we discuss the most pertinent remaining gaps from an environmental risk perspective, commenting on the importance of continuous improvement in legislation to move towards the use of such knowledge.

### Risk assessment of mixtures

Pharmaceuticals do not exist in the environment as individual compounds but as a part of mixtures, co-occurring alongside other pharmaceuticals, their transformation products and other potentially harmful chemicals. Monitoring schemes in a range of European countries (e.g. France, UK, Portugal and the Netherlands, as well as across the River Elbe catchment in central Europe) have identified the presence of multiple pharmaceuticals in environmental media, such as surface water and groundwater. These mixtures include a range of chemicals, including some identified as having the greatest potential to cause effects in aquatic ecosystems [61, 62]. Some pharmaceutical products can be manufactured as complex formulations, where the active pharmaceutical ingredient is only one of several components that may cause risk in different ways, and some can be formulated as combinations of active ingredients—such products effectively represent mixtures in their own right. Hence, when considering the potential for mixture exposures, both unintentional and intentional mixtures need to be considered as realistic scenarios.

Given the prevalence of different types of combined exposures, there is a recognised need to consider how these mixtures may affect ecosystems. The basis of an assessment of mixtures containing pharmaceuticals can leverage the principles of mixture toxicology, where the dominant paradigm is that the joint effects of chemicals are characterised by additivity governed by the similarity or dissimilarity of their mechanism of action [63]. Within mixture assessment, perhaps one of the greatest challenges is to predict those cases (perhaps 10% of mixture combinations) where synergism or antagonism occurs, with synergism being the effect of greatest concern for risk assessment [64, 65], or where the prevalence of synergism and/or antagonism has a complicated dependence on the concentration, ratios and number of other chemicals present [66]. Studies of the mechanisms of synergism have begun to link some types of chemical effects, notably co-exposure with chemicals that alter toxicokinetic mechanisms, as one of the major causes of such effects. For example, exposure to azole fungicides, which are commonly used in both medical antifungal products and as pesticides, has been linked to the inhibition of cytochrome P450s, leading to synergism [67, 68]. There is also the case of pharmaceuticals designed with two or more active ingredients to achieve synergistic therapeutic effects or to enhance the product’s pharmacokinetic properties. Mixture toxicity studies should, therefore, take into account combinations of active components known to interact synergistically. For example, sulfamethoxazole and trimethoprim are the two active ingredients in the antibiotic co-trimoxazole, combined to produce a stronger antibacterial effect through synergistic inhibition of folate synthesis, which is essential for bacterial DNA replication and growth. However, this combination has been shown to cause acute synergistic effects in freshwater microalgae [69]. When such interactions are known or suspected, the potential for synergistic effects should be considered within the mixture modelling framework to include non-additive effects in the risk assessment.

Currently, mixture assessment presents a significant challenge to the regulatory system in the sense that ERAs (and other such assessments of risk) are almost always performed for individual products. Indeed, comments received on the 2024 ERA Guideline [4] regarding mixtures received responses from the EMA that, for multi-component products, “the regulatory ERA is product based, and thus it is impossible to test/predict environmental risks for all possible [active substance] combinations”, and that considering broader mixtures was “beyond the scope of the guideline revision”. Mixture assessment would present the need for manufacturers to assess their products in the context of whole formulations in the case of planned mixtures and likely co-exposures for incidental environmental mixtures. One proposed approach by the European Commission is to introduce the use of Mixture Assessment (or Allocation) Factors (MAFs) into REACH Annex I [70], which are adjustment factors applied to risk assessments to account for the risk from mixtures. Proposed updates from the latest CARACAL (Competent Authorities for REACH and CLP Implementation) meeting in 2025 include the use of MAFs for substances produced in quantities greater than 1000 tonnes/year by companies carrying out chemical safety assessments under REACH [71]. The development of MAFs is a developing subject, and research has shown that the use of generic MAFs applied to all chemicals does not adequately capture mixture effects for pharmaceuticals [72]. Therefore, developing targeted MAFs specific to pharmaceuticals tailored to different intended modes of action could be prudent.

Any approach to include mixtures in risk assessment raises the important point that we need data on the chemical mixtures present in the environment. Monitoring data give useful snapshots [3], but exposure modelling is needed to fill gaps and generate predictions for prospective assessments. Hence, there is a need to make sure that the modelling methods that are being developed for chemicals and pharmaceuticals will be flexible enough to be used for a range of different activities and that the outcomes lead to a better understanding of the composition of mixtures. Few exposure models currently exist that simulate multiple chemicals simultaneously [73], and to the authors’ best knowledge, none have so far been applied to pharmaceuticals. Assuming that chemicals behave independently in the environment obviates this need for mixture-capable models (exposure models can be run independently for different chemicals), but this is not true where chemicals influence each other; most notably when predicting the exposure of a parent compound and its transformation products.

### More holistic effects testing and prediction

Historically, regulatory ecotoxicity assessments have been weighted heavily towards conventional assessment endpoints such as survival, growth and reproduction, measured in tests performed over limited time durations, in most cases a fraction of the organisms’ full life cycle. This approach could miss important sub-lethal effects, long-term impacts and modes of action that may have significant effects on populations, communities and ecosystem services. A growing body of scientific literature is providing evidence that low-level chronic exposures over extended time-scales may affect physiological traits via distinct mechanisms of action [74, 75]. Additionally, conventional ecotoxicity studies do not capture more subtle endpoints, such as behavioural effects, which are more likely to be associated with chronic exposures [76–78].

Pharmaceuticals have specific modes of action that target physiological functions linked to aspects such as neurological functions, inflammation pathways, metabolism and endocrinology. Because they have such targets, it is reasonable to anticipate that as a group, pharmaceuticals may be more likely to have effects on non-conventional endpoints than other classes of environmental pollutant. These non-conventional endpoints do not cover parameters measured in standards or guidelines (e.g., from OECD or ISO, as survival, growth or reproduction) and are related to behaviour or molecular or cellular events. When non-conventional endpoint effects occur, this can plausibly lead to changes in individual performance and vital rates, which may propagate onto population, community and ecosystem impacts. It is welcome that the 2024 ERA Guideline [4] mentions the potential future inclusion of behavioural endpoints in tailored testing strategies for neuroactive substances when standardised guidelines become available. More specifically, it is also welcome that endocrine disruption is increasingly seen as a conventional endpoint and that flagging for endocrine disruption is required in the proposed legislation.

#### Moving beyond short-term testing on conventional endpoints

Recent work on biologically active chemicals (pesticides, biocides, pharmaceuticals) has identified a range of modes of action that may not directly affect conventional apical effects (survival, growth, reproduction), but may affect other aspects of physiology [79, 80]. Examples of such mechanisms are neurotoxicity (affecting behavioural endpoints), immune modulation (affecting disease/parasitism vulnerability), genotoxicity (affecting offspring quality or other fitness parameters) and metabolism-related responses (affecting energy budgets). These types of effects are identified as being more likely to occur during chronic exposures [81]. Effects under such low-level, chronic exposures can be subtle and are often challenging to locate, characterise and quantify—particularly in a regulatory context where lengthy and costly experiments to generate data could be considered as a barrier for industry. Integrative approaches are needed to mechanistically link complex chronic exposures to the subtle non-lethal effects at different levels of biological complexity (from molecule to individual) to establish the causality of dose–response relationships and assess risks of chronic low-level exposures to chemicals [79, 81]. There is an argument that the extensive human safety testing required for pharmaceuticals reduces the likelihood of these effects compared to other chemicals. However, human testing may still miss chronic effects and (unintended) mechanisms of action not applicable to humans, especially as reproductive, immunological and detoxification systems have dramatically diverged between invertebrate species, and even to a functionally important extent across more closely-related vertebrate taxa. One notable example is the impact of non-steroidal anti-inflammatory drugs on kidney function leading to visceral gout in birds of prey, causing a catastrophic collapse in the vulture populations of Pakistan, India and Nepal [82, 83].

**Endocrine disruption**

Represents a pharmaceutical-relevant mode of action that can result in population-level effects [84]. As such, the proposed EU legislation recognises this by including a requirement to label whether substances are endocrine disruptors. Despite this recognition, there are some limitations to the current approach. One of the major concerns is that the measurement of endocrine disruption in other areas of chemical risk assessment, for example, for plant protection products [85], mainly considers effects through three major axes: the oestrogen, androgen and thyroid pathways. These axes are clearly crucial for their effects on maturation and reproduction for oestrogen, and androgen and for development (e.g. metamorphosis in amphibians) and some aspects of metabolism. That said, these three pathways are, however, not the only endocrine system that chemicals can affect. Other hormone systems, for example, the corticosteroid, retinoic X and PPAR (peroxisome proliferator-activated receptor) pathways also act as critical controls of physiological responses, including development and metabolism. Chemicals have been shown to interact with these pathways, leading to biological effects [84, 86, 87]. Hence, as evidence develops, there may be a need to consider a wider range of endocrine effects within assessments. The use of a biosensor method that allows an assessment of whether a substance can act with each of these hormone receptors (usually using the human variant), would be a first step to understanding how frequent endocrine activity through these additional pathways may be [88, 89]. The yeast oestrogen and androgen screening (YES/YAS) assays [90] serve this purpose for oestrogen and androgen pathways, and their use and development opens the door for further development to cover other important pathways.

A further issue for the holistic endocrine assessment across taxa is that the current high-throughput methods used to test for disruption are biased towards vertebrate (particularly mammalian) endocrinology [91, 92]. For example, insects are generally accepted to lack oestrogen or androgen systems, instead having systems linked to ecdysteroids and juvenile hormones. Hence, conclusions on endocrine effects on steroid hormone systems are not likely to be relevant for a group like insects that lack these systems. Screening pharmaceuticals for potential endocrine disruption in invertebrates will require the development of suitable high-throughput methods and assays that target pathways relevant to different taxa. Currently, despite some real progress [93], our capacity to develop such assays is inhibited by a lack of knowledge of endocrinology in some important invertebrate groups, highlighting the need for a greater focus on comparative invertebrate endocrinology [94].

#### Behavioural change

Effects on behaviour through neuronal system interactions is a further example of a potential impact relatively poorly addressed by conventional ecotoxicology testing. Effects on neuronal pathways leading to behavioural change were, for example, found to be critical in the impact of neonicotinoid insecticides on pollinators [95]. In a more recent study, it was demonstrated that the anxiolytic clobazam is globally present, accumulates in the brain of exposed fish and influences river-to-sea migration success [96]. These concerns have led to an increasing interest in behavioural ecotoxicology. For example, a survey of data on the US-EPA Knowledge Base (covering data from 2000 to 2024) suggests *Apis mellifera* is the fourth most studied species (in terms of ecotoxicology) by comparison of total entries (combining chemicals, all effects and all endpoints). Within this, evidence-based, behavioural assays are a non-trivial component (~ 23% of all entries), with 45 distinct behavioural endpoints measured. Outside of bees, behaviour is an under-represented aspect in ecotoxicological research (e.g., behavioural assays represent only ~ 3% of all US-EPA Knowledge Base entries over the same period for *Eisenia fetida*, the earthworm of choice for ecotoxicology). The relevance and interest in behavioural effects in pollinators point to the need for developing reliable behavioural assays for a greater range of invertebrates, especially, but not only, those expected to show complex behaviours such as courtship, territoriality, migration, etc. [78]. The OECD has already reflected a focus on the potential for behavioural effects by increasing the inclusion of some element of behaviour into a number of assays [97].

For pharmaceuticals, the need for behavioural assays is reinforced by mounting evidence that pharmaceutical-induced behavioural effects are observed at realistic environmental concentrations and so would require consideration in regulation. For example, antidepressants have been shown to have behavioural effects on a range of species at realistic environmental concentrations [98–100]. A range of such behavioural assays have been proposed by Peterson et al., [101], and methodological and technological innovations in recent years, such as object tracking in videos powered by machine learning, have boosted our ability to collect high-resolution and broad-scale data on behavioural changes [76]. One notable example is the development of a high-throughput screening assay using *Daphnia magna*, measuring non-associative short-term memory and learning responses, such as sensitisation and habituation to repetitive light stimuli [102].

In the terrestrial compartment, there are already behavioural endpoints in standards assessing soil quality, such as ISO17512 (Parts 1 and 2) [103, 104], which includes avoidance tests for determining chemical effects on behaviour in earthworms and collembola, respectively. Using this standard, springtails (collembolan *Folsomia candida*) exposed for a short period of time (48 h) to carbamazepine presented an avoidance behaviour, while for fluoxetine exposure this did not occur [105]. Although both compounds have different mechanisms of action in humans, these results were unexpected, considering that both have a mode of action related to the nervous system. Following these results, a multigeneration test further explored changes in springtail behaviour exposed to carbamazepine and fluoxetine and observed that *Folsomia candida* 3rd generation juveniles (F3) lost their phototaxis typical behaviour, losing the ability to escape from light [106]. Again, this was more stressed in the carbamazepine multigenerational exposure than for fluoxetine. However, whilst this field is rapidly advancing, there is a need to generate guidance towards how such behavioural data may be used in ERA [97].

Part of the argument for including behavioural effects in ERA is that it is possible to link effects to impacts on populations. As the case of pollinators exposed to neonicotinoids has shown, behaviour change can be linked to population status through effects on navigation and foraging efficiency [107, 108]. Because of the recognised potential for ecological relevance, there is growing recognition of the importance of behavioural effects and acknowledgement of the difficulties behavioural testing might bring. In a recent survey of environmental scientists working in the fields of environmental toxicology and behavioural ecology, 68% agreed that regulatory authorities should consider behavioural endpoints, though 78% acknowledged that doing so would likely lead to increased costs to regulators and industry [78].

#### Mechanistic effects modelling

Mechanistic effects models can be broadly defined as tools enabling the prediction of chemical effects on organisms and ecosystems based on some kind of mechanistic conceptualisation of these effects [109]. Mechanistic models can provide a key way of linking exposure and effects data to predict impacts on ecosystems. This sets them aside from in silico tools, which are mainly empirical and driven by statistical fits, rather than parameterised mechanisms.

At the organism level, mechanistic toxicokinetic/toxicodynamic (TK/TD) models simulate a given effect endpoint over a period of time using information on the underlying process of chemical uptake and internal effects that together lead to toxicity. TK/TD models offer a solution for predicting toxicant effects beyond the experimental conditions at any time, including complex and time-varying exposure profiles, by explicitly focusing on the temporal processes and effects. In the context of pharmaceuticals, a particular interest is the use of TK/TD models to predict sub-lethal effects from chronic exposure to relatively low doses, though the development of such models is currently sparse [109, 110]. Most TK/TD model applications to this end are based on approaches within the dynamic energy budget (DEB) family of models that either include a TK/TD component, to model effects on multiple endpoints, or are limited only to effects on mortality prediction using the General Unified Theory of Survival (GUTS) component [111]. Because it addresses effects across different (multiple) apical endpoints, DEB TK/TD-based models can be used to provide data from which to predict the population-relevant consequences of environmentally realistic pharmaceutical exposure scenarios.

Outside of the widely used DEB-based set of tools, a host of other population-level models can potentially be used to make this link between exposure and effects, ranging from simple exponential or logistic growth to more complex individual-based models that account for organism variability [109]. Combining different modelling approaches can also be used to link individual and population effects, such as coupling DEB-based models with individual-based models [112]. Such approaches are appealing as they allow for assessing population-level effects based on commonly measured endpoints used during regulatory assessment (e.g. survival, growth and reproduction). However, whilst such models are established, there is a clear need for future work to expand and validate the use of mechanistic effects modelling for pharmaceutical ERA.

#### *In-silico* prediction and new approach methodologies

The large number of pharmaceutical compounds, diversity of potentially exposed species, and a desire to reduce animal testing make New Approach Methodologies (NAMs) an attractive solution for use in pharmaceutical ERA. NAMs comprise a range of innovate methods to assess risk without relying on animal testing, including in vitro and in silico assessments and omics. Taking the example of in silico assessment, a range of long-standing predictive approaches are being applied to assess the environmental risks posed by pharmaceuticals [113], e.g., Quantitative Structure–Activity Relationship (QSAR) models [114]. Critically, the detailed knowledge of pharmaceutical mechanisms of action (for target taxa) also creates the possibility of utilising ever-expanding genomic data (e.g., the Darwin Tree of Life, DTOL, consortium [115]) to identify and compare potential receptors across non-target taxa. This effort has been pioneered by the development of multiple in silico tools. For example, the ECOdrug tool connects specific pharmaceuticals to their putative targets across divergent species [116], supporting intelligent ERA by ensuring consideration of appropriate species. Although revealing the presence of a target receptor is a critical step, it is also possible to characterise more detailed receptor differences that influence chemical interactions and, by extension, the sensitivity of environmental taxa. To this end, the SeqAPASS tool compares receptor sequences from untested species with equivalent receptors possessing well-characterised toxicant interactions [117, 118]. Specifically, SeqAPASS considers sequence homology across receptor sequence and combines this with information on the identity of specific amino acids vital to chemical interactions, thereby generating a likelihood estimate that orthologous receptors across untested taxa will interact with a given compound.

Although receptor presence and sequence are likely to be a critical factor in determining pharmaceutical-induced toxicity, other toxicodynamic and toxicokinetic factors will also contribute. The fish plasma model was developed to assess the environmental risk of pharmaceuticals and personal care products in aquatic systems by utilising pharmacokinetic and pharmacodynamic data from human drug development. It compares human therapeutic plasma concentrations with predicted steady-state concentrations in fish, assuming drug target conservation across species [119]. While the assumptions of steady-state conditions and shared toxicokinetics between humans and fish are key limitations, the fish plasma model is a useful screening tool for prioritising pharmaceuticals for risk assessment [120]. The Adverse Outcome Pathway (AOP) concept expands the toxicodynamic dimension by connecting chemical-receptor interactions with a series of downstream events required to cause an eventual adverse outcome [121]. The adverse outcome can be environmental in nature. For example, putative AOPs exist that detail interactions between pesticides and their receptors in environmentally critical species, leading to population-level effects via multiple levels of biological organisation [122]. As such, AOPs provide a potentially quantitative [123] approach to linking chemical exposure to the mechanistic triggering of biological effect and its apical consequences. The in silico G2P-SCAN tool [124] aims to exploit well-characterised AOPs and expand genomic resources to perform species comparisons across a broader range of AOP-linked molecular components, taking predictions beyond just chemical-receptor interactions. Recent work has begun the process of combining these in silico new approach methodologies into a more holistic predictive framework for predicting cross-species chemical susceptibility, with a clear effort to include pharmaceutical-relevant target receptors and AOPs [125].

Still, the use of NAMs to fully replace animal testing carries certain limitations in achieving complete in vivo predictability, especially when it comes to complex processes like developmental and reproductive toxicity, pharmacokinetics and metabolism or mixture toxicity [126]. Such a transition requires a robust framework for in vitro to in vivo extrapolation (IVIVE), developing and validating specific sets of test batteries and having a comprehensive mechanistic understanding, in this case, of pharmaceuticals [127].

### Better accounting for environmental heterogeneity

The simple calculations or screening-level exposure models most often used in ERAs lack fidelity to the real world and its heterogeneity. They provide conservative estimates of average concentrations, often across large (e.g. national) scales. The reliance on these models is based on pragmatism: they are minimal in their data requirements and, therefore, relatively easy to use, and by making conservative assumptions they can obviate the need for more realistic, higher-level exposure predictions. Their results are often easier to understand and interpret than the (uncertainty) distributions provided by more complex models. However, as modelling paradigms and spatial data availability advance, it is worth reassessing whether this trade-off is still sensible or whether there is a need for higher-tier models that might offer spatiotemporal predictions and more robust conceptualisations of the chemistry affecting pharmaceutical compounds in the environment.

Before advocating a move towards higher accuracy, at the expense of higher complexity, it is first worth considering whether such a move is required or justified. More complex models require more data, are more difficult to operate, and their results are more difficult to interpret. Whilst these issues can be tackled (as discussed later), there is a risk that such a move will result in more lengthy and costly assessments that are more difficult to interpret. Furthermore, added complexity does not necessarily mean increased environmental protection through better understanding. Even if there are the data to make such assessments, there is always the risk of unexpected exposure or effects that are not considered in our current assessments. A notable example already given above is that of diclofenac poisoning vultures in Pakistan, India and Nepal [82, 83], but there are numerous other examples. Given that a fundamental pillar of risk assessment is protection, there is a strong argument for simpler assessments, such as the previously discussed MAFs or other adjustment factors, which can account for unexpected exposures and effects, at the same time as keeping assessments timely, manageable and understandable, both to regulators and wider society [128]. Moving towards more complex assessments should only be pursued when we have implemented approaches such as MAFs and accounted for non-convential effects (e.g. behavioural) to ensure that our environmental risk assessments are protective.

**Fig. 2:**
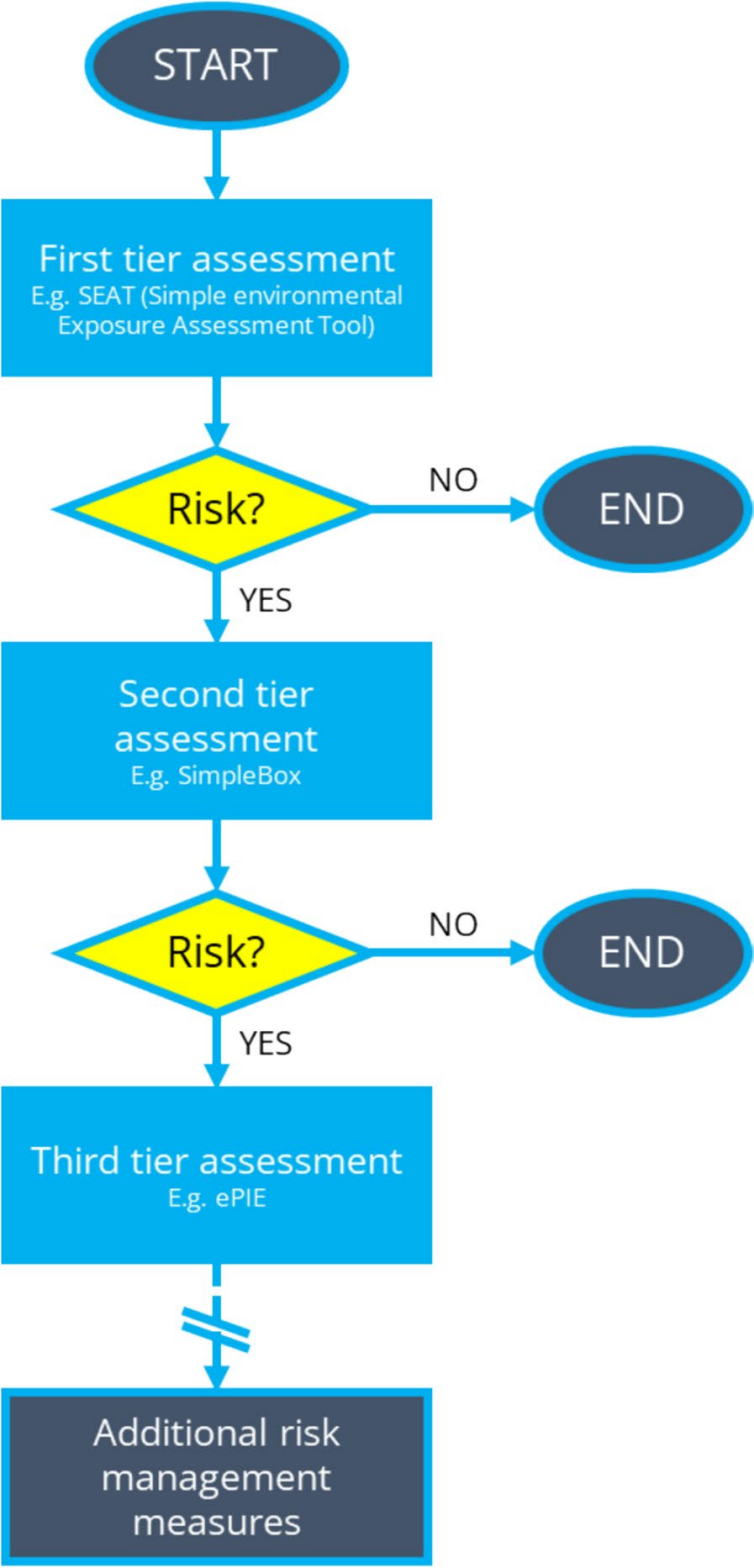
An example tiered exposure assessment for pharmaceuticals. The assessment only progresses to the next tier if the calculated risk is significant enough, otherwise the assessment ends and the product is deemed unlikely to pose a risk to the environment. Example models are given for each tier: SEAT (Simple environmental Exposure Assessment Tool) [132]; SimpleBox [133] and ePIE [134]

In the context of regulatory risk assessment, a tiered exposure assessment framework is most useful. The current ERA guideline [4] already takes a phased approach to risk, requiring determination of additional fate and hazard parameters for the higher phase. However, the PEC calculation remains relatively simplistic for all phases/tiers, and there are benefits to taking a tiered approach to exposure assessment. Under a tiered exposure assessment framework, products requiring market authorisation first undergo a screening-level (first tier) ERA, similar to the current guideline. This assessment is conservative, and thus, if the PEC is much lower than a relevant effect concentration or a regulatory PEC action limit, then it can be concluded that the product is unlikely to pose a risk to the environment. However, if the PEC approaches (or exceeds) this concentration, then a higher-tier assessment could refine the exposure assessment to explore whether the conservative assumptions in the first tier are appropriate, or whether targeted modifications to the product (lifecycle) could reduce the risk to a level deemed acceptable. An example framework is depicted in Fig. 2. Within regulation, the first tier would be mandatory, whilst the higher tiers would only be employed if the manufacturer believes that a more detailed assessment will demonstrate that their product is safe. Tiered exposure assessments are already employed in the risk assessment of plant protection products in the EU [129] and have been proposed for other substances such as nanomaterials [130], though this implementation is not without its difficulties (e.g., the risk of higher-tier models underpredicting exposure concentrations [131]). A similar framework could be beneficial for pharmaceuticals.

A tiered exposure assessment offers several potential advantages. For example, refining the PEC calculation in higher tiers might lower PECs enough to give confidence that further studies assessing fate and ecotoxicity parameters are not required (essentially, completing a tiered exposure assessment under the current Phase I might mean the manufacturer does not have to move on to Phase II). Aside from potentially making the assessment less costly, this could reduce the reliance on animal testing for ecotoxicity studies.

One scenario where there are likely to be significant differences between low- and high-tier models is when there are highly local emissions or other local factors affecting exposure. Current low-tier exposure models may fail to capture local peaks in pharmaceutical concentrations, which should, in theory, be counteracted by their conservative assumptions. However, this design offers manufacturers limited information on how mitigation or management measures may be best targeted to reduce risk, making it difficult to assess whether low-tier assumptions are sufficiently conservative so as not to underestimate risk in contamination hotspots. Enhanced, user-friendly spatiotemporal exposure models that consider local variations and extremes could help resolve these issues. Such models should provide more realistic assessments by incorporating local environmental conditions and accurately predicting exposure dynamics. Issues around the complexity of input data required for these more spatially resolved models should be solved by pre-providing specific geospatial data (e.g., hydrological flows for reference periods, soil texture from data sources like the European Soil Data Centre) and providing a map-based user interface for users. Regulators could collate these pre-defined input datasets to provide a standardised way for manufacturers to perform high-tier assessments without the complexities of spatiotemporal data collation. The FOCUS (Forum for Co-ordination of pesticide fate models and their USe) models and scenarios for plant protection products is a good example of how such harmonisation of models and scenarios can facilitate the use of high-tier models in regulation [135], though there are notable difficulties in, for example, the relevance of existing scenarios, the proprietary nature of the models used, and the accuracy of higher-tier models [131]. Further improvements in model accessibility beyond this could include hosting the model and interface on a scalable cloud server, which could alleviate issues around computational demand. Investment would be needed in both areas as developing user interfaces and hosting on cloud servers can be time-consuming and costly.

Developing pharma-specific models is also a valuable approach. Existing models like SimpleBox [133] do not fully address the unique behaviours of pharmaceuticals, particularly polar compounds. Pharma-specific models, such as ePIE, need to be developed and refined to account for the distinct properties of pharmaceuticals, including their ionization and interaction with organic matter, as well being able to track their transformation products. Ideally, these models would be multimedia, considering the full fate pathway through at least aquatic and terrestrial environments. These tailored models can provide more accurate predictions of pharmaceutical fate, exposure, and uptake in various environmental contexts. Furthermore, understanding chemistry and bioavailability is also important. Environmental factors such as pH and organic matter content significantly influence the behaviour, bioavailability and impact of pharmaceuticals [136]. Current risk assessments could incorporate these medium-specific factors to improve accuracy, potentially enabling targeted mitigations or reduce the need for further testing. Advances in in silico approaches, such as Quantitative Structure–Activity/Property Relationship (QSAR/QSPR) models, can aid in predicting the environmental behaviour of pharmaceuticals by calculating extrinsic fate- and effect-relevant properties from intrinsic properties such as molecular structure and how this modifies medium-specific behaviour [114, 137, 138]. Robust QSAR/QSPRs reduce the reliance on costly and time-consuming laboratory experiments to deduce extrinsic parameters, at the same time enabling better resolution across relevant intrinsic and media properties, though, as previously discussed, caution is needed in extrapolating ex vivo results to in vivo predictions [126, 127].

Pharmaceuticals exist in the environment as part of mixtures with other contaminants. The importance of accounting for mixture effects in ERA has already been discussed, but it is worth reflecting here that understanding mixture effects requires real-world data on mixture exposures, which are likely to be highly heterogeneous across different environments [3, 139]. This information could be gained from data to inform mixture-capable exposure models (e.g., emissions data of various contaminants) or observations that measure multiple compounds in appropriate locations. Developing mixture-capable exposure models is relatively straightforward; the difficulty comes in sourcing real-world data on use, release and environmental behaviour. Fortunately, initiatives like the NORMAN Database System [139], alongside various national monitoring schemes, are increasing the abundance of such data, particularly for surface waters. Extending the availability of terrestrial mixture exposure data is essential for ensuring that mixture assessment covers the most relevant environmental compartments.

### Limiting environmental emissions

So far, our discussion has focussed predominantly on exposure and effects, but ultimately, the emissions of pharmaceuticals from different lifecycle stages is the key driver of environmental risk. Limiting emissions is an effective way at reducing risk, and legislation should play a role here. Therefore, it is notable that the updated legislation makes little mention of emissions, except that “The applicant shall also include in the ERA risk mitigation measures to avoid or where it is not possible, limit emissions to air, water and soil” (Directive Article 22.3 [10]).

Biosolids application and wastewater irrigation are key emission pathways for pharmaceuticals [140, 141]. There is separate legislation in the EU that covers these, namely the EU Sludge Directive [142] and Water Reuse Regulation [143], and a full assessment of this legislation is beyond the scope of this paper. However, it is worth noting how, if at all, pharmaceuticals are considered in this legislation. On biosolids, pharmaceuticals are not explicitly mentioned in the Sludge Directive, and the only contaminants for which there are defined threshold values are metals. Thresholds for pharmaceuticals could be introduced to reduce risk to soils from pharmaceutical pollution, or the EU could consider the more politically contentious route of restricting the application of biosolids (several countries, like the Netherlands and Switzerland, already heavily restrict biosolids application). On wastewater reuse, though there are no water quality requirements specifically for pharmaceuticals (Water Reuse Regulation [143]), the Regulation does explicitly state that further mitigation measures must be considered if risk arises due to other contaminants, including pharmaceuticals, “when there is clear scientific evidence that the risk originates from reclaimed water and not from other sources” (Annex II (B) 6).

A significant amount of medicines enter the environment due to down-the-drain disposal by patients, and has been much discussed elsewhere, tackling this improper disposal is key to limiting emissions [144]. It is therefore welcome that the updated legislation requires labelling including “specific precautions relating to the disposal of unused medicinal products … as well as reference to any appropriate collection system in place”. However, for this to be effective, it needs to be backed up by clear communication campaigns to the public, ideally supported by the pharmacists and outlets who sell over-the-counter pharmaceuticals.

## Discussion

Here, we have reflected on the proposed update to pharmaceutical legislation in the EU, offering thoughts on how pharmaceutical legislation could be continuously strengthened towards environmental protection.

A key question that arises from this discussion is what strategy should be employed to enable this continuous improvement, ensuring that regulation keeps pace with scientific knowledge. Traditionally, legislative updates have been sparse and often delayed by lengthy consulation periods, but relatively major when they occur, like the new update for pharmaceuticals. Regulatory changes can significantly impact the industry, requiring long-established practices and workflows to be changed to gain regulatory approval. Updating regulations also requires significant effort to develop them and consultation with stakeholders when drafted, which is both costly and time-consuming. These factors naturally limit the frequency of regulatory updates, creating a potential lag in scientific knowledge. An alternative paradigm could be a shift towards iterative regulatory development, whereby smaller incremental changes are made over shorter timeframes. This means that regulation would be able to keep pace with scientific understanding better, but it could be seen as “shifting goalposts” where the industry must keep on top of changes constantly. There is an argument that smaller, more frequent changes could be less disruptive for the industry than larger, infrequent changes. There is likely a sweet spot between the two, perhaps consisting of a relatively static overarching framework laying out the principles and requirements of regulatory assessment, but within which changes can happen in a streamlined process. These streamlined changes could include recommendations for exposure modelling approaches or methods to deduce relevant effect endpoints (and perhaps even what effect endpoints are required).

Furthermore, a process similar to a mutual recognition procedure for sharing ERA data—such as a publicly accessible database managed by the EMA—was proposed by a public consultation as a key facilitator for new marketing authorisations, enabling broader data sharing and improved stakeholder accessibility. Harmonising ERA procedures and protection goals across regulators in different member states is essential to prevent divergent outcomes based on identical medicine dossiers and to avoid time-consuming document adaptation processes [145].

Outside of environmental risk considerations, numerous other factors determine how “green” a particular pharmaceutical product is. These are often captured in LCA, including impact categories such as carbon-equivalent footprint and resource demand. It is broadly recognised that environmental risk should be a separate category within LCA [43, 45, 146], and should cover the full product lifecycle (cradle-to-grave) rather than only up to distribution (cradle-to-gate). LCA, including environmental risk across the full product lifecycle, would provide a more complete picture of how sustainable a particular product is. While such an assessment could be used to make decisions, for example, on which product formulation or manufacturing technique is preferable, the data requirements of a full LCA limit its usage at the early stages of product development. Instead, simplified LCA or other frameworks targeted at early-stage decision-making could be used, such as the Framework for Early-Stage Sustainability Assessment (FESSA) [147], which allows for multi-criteria decision analysis in the presence of uncertainty. The EU Joint Research Centre’s Safe-and-Sustainable-by-Design (SSbD) framework [148] is a compelling framework within which to perform this kind of assessment and decision-making. Recent work demonstrating the applicability of the SSbD framework to the pharmaceutical sector should be used as guidance [149].

Despite developments in this area, key questions relating to LCA and sustainability assessments remain. Notably, what weights should be applied to different criteria within this decision-making process? For example, how can societal benefits be weighed against environmental risk or climate footprint? What decision should be made if a drug has immediate public health benefits but potential long-term ecosystem effects? Clearly, there is no easy answer and balancing these different criteria is highly subjective. Results from a public consultation highlighted concerns about incorporating ERA outcomes into the benefit-risk assessment, emphasising that the primary focus should be on ensuring the availability of the medicines for patients rather than prioritising environmental safety [145].

By broadening the perspective on pharmaceuticals, we raise the issue of responsibility. This is particularly true when considering mixtures. If a pharmaceutical product is considered environmentally risky due to its occurrence alongside other chemicals, is it the manufacturer of the pharmaceutical or the other chemicals that bears the responsibility for the risk? While using MAFs can offer protection against risks arising from mixtures, it does not solve the issue of how responsibility for risks is shared amongst manufacturers [150]. Environmental mixture exposure is highly variable and strongly dependent on legacy emissions and waste streams, particularly in compartments that act as sinks, such as soils and sediments. It is often impossible to deduce who (e.g., what product) is responsible for what proportion of a given chemical exposure. Given this lack of connection, it would be very challenging for regulatory authorities to share the responsibility of mixtures amongst manufacturers fairly. The burden of mixtures also falls across regulatory regimes. For example, mixtures with components from different sources, such as a pharmaceutical compound, a plant protection product and a PFAS compound, would, in the EU, fall under the remit of the EMA, European Food Safety Authority (EFSA) and European Chemicals Agency (ECHA).

At all levels, there is room for better integration. At the assessment level, exposure and hazard assessments are often performed separately, with results combined in a final step to calculate overall risk. By better integrating these approaches, it would be easier to include the influence of drivers that affect both exposure and hazard, such as temperature and pH. Ideally, this integrated assessment would include mixture exposures and effects. Such an approach has already been taken for plant protection products [73]. As previously discussed, better integration between regulatory regimes is crucial at the regulatory level. The European Commission’s “one substance, one assessment” initiative is a welcome step in this direction.

Transformation products of pharmaceuticals include metabolites excreted by humans and animals, and by-products formed during degradation processes in wastewater treatment and the environment. These compounds have been detected in the environment and often demonstrate equal or greater toxicity to biota than their parental compounds. However, they are typically not considered in the preliminary exposure assessment of ERA for pharmaceuticals [151, 152]. This issue is not explicitly addressed in the Directive or Regulation but is mentioned in the updated EMA guideline (2024). The guideline states that if “the active substance is completely excreted as parent compound without metabolism or assuming that metabolites have similar or lower toxicity than that of the parent compound”, a “total residue approach” should be applied. This means metabolites are treated as their parent compounds, and their ERA is considered covered. If this assumption is not met, a full Phase II risk assessment must be conducted for metabolites [4]. This would significantly increase the overall ERA cost by including both the parent compound and individual metabolites. This, combined with the lack of environmental fate and ecotoxicity data for most metabolites, will likely lead to a preference for the total residue approach [153]. In this context, in silico tools, such as QSARs, could provide valuable predictions regarding the environmental fate and effects of metabolites or degradation products. However, experimental data on these substances remains essential for ensuring a comprehensive assessment.

Despite the significant progress made in the area of NAMs, there are still limitations in terms of validation and regulatory decision-making [154]. Research on NAMs for pharmaceutical ecotoxicological assessment should be prioritised. Every year in the EU, more than a million fish are used for scientific purposes, turning it into an ethically questionable and controversial approach [155]. Developing high-throughput in vitro screening assays to characterise uptake and ADME (absorption, distribution, metabolism and excretion) properties of pharmaceuticals in fish species (e.g., zebrafish, rainbow trout)will facilitate the generation of large datasets for predictive modelling to obtain ADME profiles and extrapolate them across species [156]. Rivetti et al. [154] suggest the use of a robust framework that accelerates the use of NAMs for environmental risk assessment through integration of conventional data via a Weight of Evidence approach, showing that it may provide protective decisions towards environmental and, eventually, human health assessment.

We acknowledge that the new measures required to comply with the updated ERA assessment for pharmaceuticals may impose additional burdens on the industry. Many companies, particularly smaller ones, may lack the resources or expertise to conduct environmental assessments (e.g., ecotoxicity studies). To address this challenge, collaborative efforts with academic institutions through initiatives like joint projects should be emphasised. These collaborations can provide companies with the necessary expertise and data for conducting comprehensive ERA assessments. For instance, EU projects like those within the EU Green Pharma Cluster [40, 48] (Enviromed, ETERNAL, Impactive, SusPharma and TRANSPHARM) foster partnerships with stakeholder organisations to conduct ecotoxicological evaluations of their pharmaceutical products (e.g., testing the ecotoxicity of APIs, solvents and other substances), promoting sustainable approaches to pharmaceutical manufacturing.

Finally, though the focus of this publication has been on pharmaceuticals, many of the points discussed apply broadly across different chemicals and regulatory regimes. Similarly, many examples from other regulatory regimes could be used as the foundations for strengthening pharmaceutical regulation. In this publication, we have highlighted several aspects of plant protection product regulation that could be used as inspiration. There is a clear need for cross-sectoral and multi-agency discussions to maximise these shared opportunities.

## Conclusion

In this publication, we have reviewed the new proposed EU pharmaceutical legislation from the perspective of environmental risk. Broadly speaking, the proposed legislation offers strengthened environmental protection. However, there are several areas in which integrating the latest scientific knowledge could further strengthen environmental protection. We discussed these areas, including mixture assessment, non-conventional and chronic endpoints, endocrine disruption, mechanistic effects and in silico modelling, and the influence of environmental heterogeneity. Finally, we discussed what these improvements would mean for regulation and placed them in the broader context of environmental sustainability throughout a pharmaceutical product’s entire lifecycle.

## Data Availability

No datasets were generated or analysed during the current study.

## Abbreviations

ADME: Absorption, distribution, metabolism and excretion
AMR: Antimicrobial resistance
AOP: Adverse outcome pathway
API: Active pharmaceutical ingredient
DEB: Dynamic energy budget
EMA: European Medicines Agency
ERA: Environmental risk assessment
EU: European Union
LCA: Life cycle assessment
MAF: Mixture assessment/allocation factor
PEC: Predicted environmental concentration
PMT/PBT: Persistent, mobile/bioaccumulative and toxic
QSAR/QSPR: Quantitative Structure–Activity/Property Relationship
SSbD: Safe-and-Sustainable-by-Design
TK/TD: Toxicokinetic/toxicodynamic
vPvM/vPvB: Very persistent and very mobile/bioaccumulative
